# Oral Human Papillomavirus (HPV) Infection among Unvaccinated High-Risk Young Adults

**DOI:** 10.3390/cancers6031691

**Published:** 2014-08-14

**Authors:** Gypsyamber D’Souza, Nicole Kluz, Alicia Wentz, Renee M. Youngfellow, Anne Griffioen, Emily Stammer, Yingshi Guo, Weihong Xiao, Maura L. Gillison

**Affiliations:** 1 Johns Hopkins Bloomberg School of Public Health, Baltimore, MD 21205, USA; E-Mails: nkluz1@jhu.edu (N.K.); awentz2@jhmi.edu (A.W.); anniegriffioen@gmail.com (A.G.); estammer@gmail.com (E.S.); 2 Baltimore County Health Department, Baltimore, MD 21212, USA; E-Mail: ryoungfellow@baltimorecountymd.gov; 3 Ohio State University Comprehensive Cancer Center, Columbus, OH 43202, USA; E-Mails: Yingshi.Guo@osumc.edu (Y.G.); Weihong.Xiao@osumc.edu (W.X.); maura.gillison@osumc.edu (M.L.G.)

**Keywords:** oral HPV, young adults, STD clinic, gender

## Abstract

Oral HPV infection, the cause of most oropharyngeal cancer in the U.S., is not well studied among high-risk young adults. Men (*n* = 340) and women (*n* = 270) aged 18–25 years attending Baltimore County STD clinics were recruited if they declined HPV vaccination. Each participant had a 30-second oral rinse and gargle sample tested for 37 types of HPV DNA, and a risk-factor survey. Factors associated with prevalent infection were explored using log binomial regression. Men had higher prevalence of any oral HPV (15.3% *vs.* 7.8%, *p* = 0.004) and vaccine-type oral HPV (*i.e.*, HPV16/18/6/11: 5.0% *vs.* 1.1%, *p* = 0.007) infection than women. In multivariate analysis, male gender (aPR = 1.93, 95% CI = 1.10–3.39), number of recent oral sex partners (*p*-trend = 0.013) and having ever performed oral sex on a woman (aPR = 1.73, 95% CI = 1.06–2.82) were associated with increased oral HPV prevalence. Performing oral sex on a woman may confer higher risk of oral HPV acquisition than performing oral sex on a man.

## 1. Introduction

While HPV vaccination is now recommended for both girls and boys in the U.S., many young adults remain unvaccinated [1,2]. Oral HPV infection, which now causes the majority of oropharyngeal cancer in the U.S., has not been well studied among high-risk young adults, although this is one of the groups most likely to transmit STDs such as HPV [3]. Existing oral HPV studies have focused on lower-risk populations such as high school/university students [4,5,6,7], or the general population [8].

Oral HPV infection is associated with sexual behavior [7,9,10], and prevalence increases with number of oral sexual partners. Some studies have suggested that oral HPV prevalence remains higher among men than women, even after adjusting for oral sexual behavior [8,11], although other studies, including a large systematic review, found no difference in oral HPV by gender [12]. Several studies included only men [4,13,14] or only women [15,16], preventing a comparison by gender.

Recent studies have reported oral HPV incidence to be higher among heterosexual than homosexual men, consistent with a hypothesis of higher risk of oral HPV transmission for cunnilingus (performing oral sex on a woman) than fellatio (performing oral sex on a man) [11,17]. Given the higher incidence of HPV-related oropharyngeal cancer among men than women in the U.S., we sought to explore gender differences in oral HPV infections. Young adults between the ages of 18–25 usually have the highest prevalence of sexually transmitted infections, and this is true for cervical HPV infections. We explored oral HPV infection among a group expected to be at high-risk for infection: young, sexually-active adults with no history of HPV vaccination who sought care at a sexually transmitted disease (STD) clinic.

## 2. Results

### 2.1. Study Population Characteristics

A total of 775 individuals were screened for the study during the enrollment period. Of these, 165 declined or were ineligible due to the following: a history of HPV vaccination (*n* = 95; 12.3%), decided to receive the free HPV vaccination when study staff explained benefits (*n* = 34; 4.4%), age outside targeted range (*n* = 21; 2.7%), or unwillingness to return for follow-up (*n* = 15; 2.0%).

The characteristics of the 610 enrolled individuals are shown in Table 1. The median age of the young men (*n* = 340) and women (*n* = 270) enrolled was 21 years (IQR = 19–23), 69.6% were Black and 82.9% were heterosexual. Current use of tobacco (42.2%), marijuana (38.7%) and alcohol (76.1%) was common. All but two participants were sexually active, and the median age of first intercourse was 14 years among men and 15 years among women. Most of these young adults had three or more sexual partners (84.4%) and had ever performed oral sex (93.9%). Forty percent had a history of a sexually transmitted disease (STD). Men reported similar numbers of lifetime oral sex partners, but a higher number of recent oral sexual partners, than women (Table 1). 

**Table 1:** Baseline demographic and behavioral characteristics, by gender.

Characteristic	Total *n* = 610	Male *n* = 340	Female *n* = 270	*p*-Value
Age in years: Median	21	21	21	0.450
18–19	162 (26.6%)	91 (26.8%)	71 (26.4%)	0.461
20–22	245 (40.2%)	130 (38.2%)	115 (42.8%)	
23–25	202 (33.2%)	119 (35.0%)	83 (30.9%)	
Race				0.813
Black	423 (69.6%)	233 (68.7%)	190 (70.6%)	
White	133 (21.9%)	75 (22.1%)	58 (21.6%)	
Other or Multi	52 (8.6%)	31 (9.1%)	21 (7.8%)	
Education				0.146
<8th grade/some high school	100 (16.5%)	55 (16.2%)	45 (16.9%)	
High school graduate or GED	255 (42.1%)	156 (46.0%)	99 (37.1%)	
Some college/college graduate	251 (41.5%)	128 (37.8%)	123 (46.1%)	
Use mouthwash every day	132 (21.8%)	74 (21.9%)	58 (21.7%)	0.960
Brush teeth ≥2 times/day	282 (46.3%)	138 (40.7%)	144 (53.3%)	0.002
Current drug use (past month)				
Cigarette use	257 (42.2%)	155 (45.7%)	102 (37.8%)	0.093
Alcohol use	462 (76.1%)	267 (79.0%)	195 (72.5%)	0.062
Marijuana use	236 (38.7%)	157 (46.2%)	79 (29.3%)	<0.001
Cocaine use	20 (3.3%)	11 (3.2%)	9 (3.3%)	0.892
Sexual orientation *				<0.001
Heterosexual	335 (82.9%)	190 (90.0%)	145 (75.1%)	
Homosexual	32 (7.9%)	16 (7.6%)	16 (8.3%)	
Bi-sexual	37 (9.2%)	5 (2.4%)	32 (16.6%)	
Ever diagnosed with a STD	234 (40.1%)	94 (29.4%)	140 (53.2%)	<0.001
Ever had genital warts	36 (6.2%)	16 (5.0%)	20 (7.7%)	0.192
Ever had vaginal, anal or oral sex	608 (99.7%)	338 (99.4%)	270 (100%)	0.21
In lifetime, number of partners performed oral sex on				0.569
None	37 (6.1%)	23 (6.8%)	14 (5.2%)	
1–2	191 (31.3%)	103 (30.3%)	88 (32.6%)	
3–5	206 (33.8%)	112 (32.9%)	94 (34.8%)	
6–10	93 (15.2%)	58 (17.1%)	35 (13.0%)	
11 or more	83 (13.6%)	44 (12.9%)	39 (14.4%)	
In lifetime, number of vaginal sex partners				<0.001
None ^	32 (5.3%)	23 (6.9%)	9 (3.3%)	
1–2	56 (9.3%)	31 (9.3%)	25 (9.3%)	
3–5	143 (23.7%)	51 (15.3%)	92 (34.2%)	
6–10	122 (20.2%)	64 (19.2%)	58 (21.6%)	
11 or more	250 (41.5%)	165 (49.4%)	85 (31.6%)	
In lifetime, number of open-mouth kissing (deep-kissing) partners				0.005
None	8 (1.3%)	5 (1.5%)	3 (1.1%)	
1–5	205 (33.6%)	101 (29.7%)	104 (38.5%)	
6–10	133 (21.8%)	64 (18.8%)	69 (25.6%)	
11–19	111 (18.2%)	71 (20.9%)	40 (14.8%)	
20 or more	153 (25.1%)	99 (29.1%)	54 (20.0%)	
In past three months, number of people performed oral sex on				0.006
None	135 (22.1%)	86 (25.3%)	49 (18.1%)	
1	326 (53.4%)	161 (47.4%)	165 (61.1%)	
2	94 (15.4%)	56 (16.5%)	38 (14.1%)	
3 or more	55 (9.0%)	37 (10.9%)	18 (6.7%)	
In past three months, number of vaginal sex partners				<0.001
None	99 (16.4%)	62 (18.6%)	37 (13.8%)	
1	253 (42.0%)	113 (33.8%)	140 (52.0%)	
2	135 (22.4%)	76 (22.8%)	59 (21.9%)	
3 or more	116 (19.2%)	83 (24.9%)	33 (12.3%)	
In the past three months, number of open-mouth kissing partners				<0.001
None	47 (7.7%)	31 (9.1%)	16 (5.9%)	
1	292 (47.9%)	134 (39.4%)	158 (58.5%)	
2	153 (25.1%)	98 (28.8%)	55 (20.4%)	
3 or more	118 (19.3%)	77 (22.6%)	41 (15.2%)	
Age at first oral sex				0.934
13 and younger	81 (14.3%)	45 (14.5%)	36 (14.1%)	
14–15	136 (24.1%)	72 (23.2%)	64 (25.1%)	
16–17	195 (34.5%)	110 (35.5%)	85 (33.3%)	
18 and older	153 (27.1%)	83 (26.8%)	70 (27.5%)	
Age at first vaginal sex				0.013
13 and younger	121 (21.3%)	81 (26.1%)	40 (15.4%)	
14–15	216 (38.0%)	114 (36.8%)	102 (39.4%)	
16–17	164 (28.8%)	84 (27.1%)	80 (30.9%)	
18 and older	68 (12.0%)	31 (10.0%)	37 (14.3%)	
Age at first sexual act				0.049
Oral sex age < vaginal sex age	35 (6.6%)	15 (5.2%)	20 (8.2%)	
Oral sex age = vaginal sex age	270 (50.6%)	137 (47.4%)	133 (54.3%)	
Oral sex age > vaginal sex age	229 (42.9%)	137 (47.4%)	92 (37.6%)	

* Only available in the subset of participants who later participated in study follow-up. ^ All except 2 of the 32 participants reporting no lifetime vaginal sex partners reported either performing oral sex (88%) and/or having been previously diagnosed with a sexually transmitted disease (44%). Most of the men and women reporting no vaginal sex partners were homosexual (*i.e.*, gay and lesbian).

The most common reasons for attending an STD clinic for both men and women were general STD testing (56.8%), STD treatment (16.0%) or STD symptoms (discharge/itching/pain; 9.1%) (Table 2). Most of these sexually active participants reported either not having a current boyfriend/girlfriend (43.2%) or having been with their current sexual partner for less than one year (32.6%). Twenty-seven percent of these young adults reported having one or more children.

**Table 2:** Reasons reported at study baseline for: (i) visiting sexually transmitted disease (STD) clinic; (ii) deciding not to become vaccinated with the HPV vaccine; and (iii) intention to become vaccinated later, by gender.

Characteristic	Total, *n* = 610	Male, *n* = 340	Female, *n* = 270	*p*-Value
**Reason for Clinic Visit**				<0.001
STD Testing	338 (56.8%)	185 (55.6%)	153 (58.4%)	
Treat STD	95 (16.0%)	69 (20.7%)	26 (9.9%)	
Discharge/itching/pain	54 (9.1%)	16 (4.8%)	38 (14.5%)	
Came with friend/partner/spouse	44 (7.4%)	25 (7.5%)	19 (7.3%)	
Heard about study from a sexual partner/friend	36 (6.1%)	23 (6.9%)	13 (5.0%)	
HIV testing	18 (3.0%)	10 (3.0%)	8 (3.1%)	
Family planning/other	10 (1.7%)	5 (1.5%)	5 (1.9%)	
**Reported reason for not being vaccinated with the HPV vaccine**				<0.001
No reason	251 (41.2%)	125 (36.8%)	126 (46.8%)	
Never heard of HPV vaccine	220 (36.1%)	157 (46.2%)	63 (23.4%)	
Worry about side effects	47 (7.7%)	10 (2.9%)	37 (13.8%)	
More research is needed/need more information	13 (2.1%)	4 (1.2%)	9 (3.3%)	
Too Expensive	12 (2.0%)	7 (2.1%)	5 (1.9%)	
I don’t have genital warts (men only)		27 (7.9%)	0 (0.0%)	
Have not had time (women only)		0 (0.0%)	20 (7.4%)	
Don’t believe it prevents cervical cancer (women only)		0 (0.0%)	3 (1.1%)	
Other ^	16 (2.6%)	10 (2.9%)	6 (2.2%)	
**Reported intention to get the HPV vaccine later**				0.005
No, not interested	123 (20.3%)	56 (16.6%)	67 (25.0%)	
Not sure	271 (44.7%)	150 (44.4%)	121 (45.1%)	
Yes, later this year	127 (21.0%)	86 (25.4%)	41 (15.3%)	
Yes, but not this year	85 (14.0%)	46 (13.6%)	39 (14.6%)	

^ Other reasons for not getting the HPV vaccine included 6 men that reported the vaccine was not available, 3 men that reported it was not important, 3 who reported that they already have HPV infection, one who reported being allergic, one who did not want any vaccine, one who had a parental concern and one who cited her pregnancy as the reason for decline.

### 2.2. Reasons for Not Getting the HPV Vaccine

Despite being within the recommended age range for HPV vaccination, many women (46.0%) and men (79.3%) reported never having discussed HPV vaccination with a healthcare provider. Additionally, only 29.9% of women and 39.0% of men reported intending to receive the HPV vaccine in the future (Table 2). As these targeted young adults were high-risk and were in the age-group recommended for catch-up vaccination, reasons for lack of vaccination were explored (Table 2). Reported reasons for not receiving the HPV vaccine included: never having heard of the HPV vaccine, concern about side effects, lack of perceived risk, lack of time, need for more information, expense, and lack of belief in vaccine efficacy.

### 2.3. Oral HPV Prevalence

Among the 610 participants in the study, 105 prevalent, type-specific oral HPV infections were detected. The most common oncogenic infections were HPV59 (*n* = 10, 1.6% prevalence), HPV16 (*n* = 8, 1.3%), and HPV51 (*n* = 7, 1.1%), and the most common non-oncogenic types were HPV6 (*n* = 9, 1.5%), HPV84 (*n* = 6, 1.0%), HPV89 and HPV66 (5 infections each, 0.8%). The baseline prevalence of oral HPV infection in the study population was 12.0% (95% CI = 9.5–14.7).

### 2.4. Risk Factors for Prevalent Infection

In univariate analysis, the prevalence of oral HPV infection was significantly higher among men than women (15.3% *vs.* 7.8%, *p* = 0.0043), as were multiple, concurrent infections (5.9% *vs.* 2.2%, *p* = 0.026), and detection of a vaccine-type oral HPV infection (5.0% *vs.* 1.1%, *p* = 0.007) (Table 3). Oral HPV16 infection was rare among both men (1.8%) and women (0.7%). Oral HPV prevalence was higher among those who performed oral sex in the past three months than those who did not (13.5% *vs.* 5.9%, *p* = 0.016). Indeed, oral HPV prevalence increased significantly with number of recent (*p*-trend = 0.008) or lifetime (*p*-trend = 0.05) oral sex partners and with number of recent (*p*-trend = 0.079) or lifetime (*p*-trend = 0.004) vaginal sex partners (Table 4).

**Table 3:** Oral HPV prevalence by gender.

Oral HPV	*n* (%)			*p*-Value
	Total	Male	Female	
BY PARTICIPANT	*n* = 610	*n* = 340	*n* = 270	
Any Oral HPV	73 (12.0%)	52 (15.3%)	21 (7.8%)	0.004
Multiple Oral HPV	26 (4.3%)	20 (5.9%)	6 (2.2%)	0.026
Any Oncogenic Oral HPV	50 (8.2%)	33 (9.7%)	17 (6.3%)	0.127
Type-16 Oral HPV	8 (1.3%)	6 (1.8%)	2 (0.7%)	0.270
Vaccine-Type Oral HPV (HPV 16/18/6/11)	20 (3.3%)	17 (5.0%)	3 (1.1%)	0.007
**Oncogenic HPV Infections**				
HPV 59	10 (1.6%)	7 (2.1%)	3 (1.1%)	
HPV 16	8 (1.3%)	6 (1.8%)	2 (0.7%)	
HPV 51	7 (1.1%)	4 (1.2%)	3 (1.1%)	
HPV 35	5 (0.8%)	3 (0.9%)	2 (0.7%)	
HPV 39	5 (0.8%)	1 (0.3%)	4 (1.5%)	
HPV 33	4 (0.7%)	4 (1.2%)	0 (0.0%)	
HPV 52	4 (0.7%)	2 (0.6%)	2 (0.7%)	
HPV 56	4 (0.7%)	2 (0.6%)	2 (0.7%)	
HPV 73	4 (0.7%)	2 (0.6%)	2 (0.7%)	
HPV 18	3 (0.5%)	2 (0.6%)	1 (0.4%)	
HPV 58	3 (0.5%)	3 (0.9%)	0 (0.0%)	
HPV 31	2 (0.3%)	2 (0.6%)	0 (0.0%)	
HPV 45	2 (0.3%)	1 (0.3%)	1 (0.4%)	
HPV 68	1 (0.1%)	0 (0.0%)	1 (0.4%)	
**Non-Oncogenic HPV Infections**				
HPV 6	9 (1.4%)	9 (2.6%)	0 (0.0%)	
HPV 84	6 (1.0%)	5 (1.5%)	1 (0.4%)	
HPV 66	5 (0.8%)	4 (1.2%)	1 (0.4%)	
HPV 89	5 (0.8%)	3 (0.9%)	2 (0.7%)	
HPV 82	4 (0.7%)	3 (0.9%)	1 (0.4%)	
HPV 53	3 (0.5%)	1 (0.3%)	2 (0.7%)	
HPV 55	3 (0.5%)	3 (0.9%)	0 (0.0%)	

**Table 4:** Univariate and multivariate risk factors for prevalent oral HPV infection.

Baseline Characteristic	*n* People	Prevalence Any Oral HPV	*n* Infections	PR (95% CI)	
				Univariate	Multivariate
**Sex**					
Female	270	7.8%	32	1.00	1.00
Male	340	15.6%	74	1.84 (1.05–3.24)	1.93 (1.10–3.39)
**Age (per year increase)**	610			1.10 (0.98–1.23)	
**Race**					
Black	423	11.8%	72	1.00	
White	133	12.0%	23	1.02 (0.56–1.85)	
Other or Multi-racial	52	15.4%	11	1.24 (0.59–2.63)	
**Current cigarette use**					
No	352	10.2%	55	1.00	
Yes	257	14.8%	51	1.27 (0.78–2.06)	
**Current boyfriend/girlfriend**					
No	263	8.4%	25	1.00	1.00
Yes	346	15.0%	81	2.47 (1.48–4.12)	2.42 (1.44–4.08)
**Current marijuana use**					
No	373	11.0%	61	1.00	
Yes	236	14.0%	45	1.17 (0.72–1.90)	
**Number of oral sex partners, last 3 months**					
None	135	5.9%	11	1.00	1.00 ^
1	326	13.2%	59	2.23 (1.02–4.88)	1.94 (0.88–4.29)
2 or more	149	15.4%	36	2.98 (1.29–6.90)	2.73 (1.20–6.25)
* p* value for trend				0.008	0.013
**Number of oral sex partners, lifetime**					
2 or fewer	228	8.3%	28	1.00	
3–19	335	14.0%	66	1.61 (0.90–2.88)	
20 or more	47	17.0%	12	2.09 (0.87–5.00)	
* p* value for trend				0.050	
**Number of vaginal sex partners, last 3 months**					
None	99	10.1%	14	1.00	
1	253	9.1%	32	0.89 (0.41–1.95)	
2 or more	251	15.5%	57	1.61 (0.79–3.27)	
* p* value (*p* value for trend)				0.079	
**Number of vaginal sex partners, lifetime**					
2 or fewer	88	8.0%	10	1.00	
3–19	362	9.1%	48	1.17 (0.50–2.73)	
20 or more	153	20.9%	45	2.60 (1.13–5.99)	
* p* value (*p* value for trend)				0.004	
Sexual orientation *					
Heterosexual	335	11.9%	63	1.00	
Homosexual/Bi-sexual	69	7.2%	6	0.46 (0.18–1.20)	
**Ever performed oral sex on a woman**					
No	335	8.1%	40	1.00	1.00 ^
Yes	275	17.1%	66	2.02 (1.20–3.38)	1.73 (1.06–2.82)
**Ever performed oral sex on a man**					
No	357	12.9%	70	1.00	
Yes	148	10.1%	21	0.52 (0.20–1.41)	
**Number of deep-kissing partners, last 3 months**					
0–1	331	10.6%	48	1.00	
2–3	205	15.6%	47	1.58 (0.95–2.64)	
4 or more	66	10.6%	22	1.14 (0.49–2.71)	
* p* value (*p* value for trend)				0.25	
**Brush teeth**					
2 or more times/day	282	11.0%	50	1.00	
1/day	291	14.1%	54	1.05 (0.64–1.72	
<daily	36	5.6%	2	0.21 (0.08–1.27)	

^ Only one sexual behavior included in model at a time. Results for recent number of oral sex partners and ever performed oral sex on a woman were each generated in separate models because of the co-linearity in these variables, but each model controlled for the same variables, as shown. * Sexual orientation was only available on a subset of participants.

As described in Table 4, oral HPV prevalence was significantly higher among men or women who had ever performed oral sex *on a woman* (*i.e*., heterosexual men or bi/homosexual women, PR = 2.02, 95% CI = 1.20–3.38). Although not significant, oral HPV infection was more common among heterosexual than homosexual men (16.8% *vs*. 6.3%, *p* = 0.18), but less common among heterosexual than homosexual women (5.5% *vs*. 12.5%, *p* = 0.20). Prevalence was also higher among those reporting a current boyfriend/girlfriend (Table 4). Race, age, current tobacco use, current marijuana use and recent and lifetime number of deep-kissing partners were not associated with prevalent oral HPV infection. 

In multivariate analysis, male gender (aPR = 1.93, 95% CI = 1.10–3.39) remained elevated after controlling for number of recent oral sex partners (*p*-trend = 0.013) and other factors (Table 4). In multivariate analysis ever performing cunnilingus (*i.e.*, oral sex on a woman) was associated with increased oral HPV prevalence (aPR = 1.73, 95% CI = 1.06–2.82), but ever performing fellatio (*i.e.*, oral sex on a man) was not (Table 4). When modeled jointly, both number of recent oral sex partners (aPR_≥2*vs.*0_ = 2.32, 95% CI = 0.95–5.76) and ever performing cunnilingus (aPR = 1.74, 95% CI = 1.00–3.01) attenuated but remained marginally significant.

## 3. Discussion

This study demonstrated a higher prevalence of oral HPV infection among men than women, even after controlling for sexual behavior. Further, it was cunnilingus specifically that was associated with oral HPV infection, in keeping with the possibility of higher risk of oral HPV acquisition during cunnilingus than fellatio [18]. These results are consistent with the hypothesis that differences in the number of oral sexual partners do not completely explain the higher risk of oral HPV infection and HPV-related oropharyngeal cancer in men compared to women [19].

The higher prevalence of oral HPV infection in men than women in this study is similar to several other recent studies performed in lower-risk study populations [5,7,8]. Indeed, several cross sectional studies of young adults suggest oral HPV prevalence of ~3%–8% in young adult men, compared to ~1%–4% in young adult women of the same age [5,8,16,20,21]. The significant gender difference found in the current study is noteworthy because most men (74.7%) and women (81.9%) had recently performed oral sex, and among these sexually active individuals a difference on oral HPV prevalence remained. Our results differ from a study of high risk young adults in Sweden, which found no difference in oral HPV prevalence among men and women, but included only 82 men [22].

Differences in oral HPV prevalence by sexual orientation have not been well explored. This study was underpowered to explore increased oral HPV prevalence among lesbian/bi-sexual women, but the findings support the need for further research on oral HPV in this group. Reporting having a boyfriend or girlfriend in this study was also associated with higher oral HPV prevalence, which may be due to the select nature of this study population enrolled in STD clinics where having a boyfriend or girlfriend may not represent a monogamous relationship.

Several barriers to receipt of the HPV vaccine observed in this study have been reported previously, including lack of information and awareness of the vaccine, concern about side effects, and lack of perceived risk for HPV infection [23,24,25]. Studies indicate recommendations from a healthcare provider can improve vaccine uptake, and we note a substantial proportion of the high-risk individuals in this study had not had such conversations. However, a large group of participants reported no specific reason for not having been vaccinated. Opt-out vaccine strategy (*i.e.*, where vaccination is given as a default to those not declining) may be particularly effective in high-risk populations such as patients at STD clinics.

This study had several strengths and weaknesses. Participants were recruited at sexually transmitted disease clinics, targeted as a group more likely to have oral HPV infection, and do not reflect the general population. Data on genital HPV infection, or HPV infections in partners of participants were not available. Strengths of this study included centralized testing of all samples, inclusion of both men and women, focus on a group at increased risk for acquisition and transmission of these infections, and collection of risk behavior information using a computer assisted self-interview (CASI).

This study demonstrated that, after adjusting for oral sexual behavior, men have a higher prevalence of oral HPV than women. This increased oral HPV prevalence among men is consistent with the higher incidence of HPV-positive oropharyngeal cancers in men than women in the U.S. [26]. Currently unknown are the relative contributions of higher infection rates *versus* longer infection duration to this difference between men and women.

## 4. Experimental Section

We enrolled young adults from two Baltimore County Health Department STD clinics between April 2010 and November 2012 in a cohort study of oral HPV infection (acronym SPITT, Study of Papillomavirus in Teens and Twenties; ClinicalTrials.gov number: NCT00994019). Patients in the targeted age range were given a study brochure when they checked into the clinic and then approached by the study coordinator if they were interested in finding out more about the study. Men and women aged 18–25 attending an STD clinic as a patient or sexual partner of a patient were eligible if they were: able to give informed consent, willing to comply with follow-up visits, able to comprehend English to complete the risk factor survey, and had no history of HPV vaccination and declined vaccination when offered. This paper analyzes the baseline results from this study. 

At study screening, all participants received counseling regarding the benefits of HPV vaccination and were offered vaccination at the STD clinic. Only those declining vaccination were enrolled.

### 4.1. Data Collection

Participants provided a 30-second oral rinse and gargle using 10 mL of Scope mouthwash to test for oral HPV DNA and completed a computer assisted self-interview (CASI) on an iPad with questions about demographics, health, lifetime and recent (past 3 months) tobacco, alcohol and drug use, and sexual behavior. Sexual behavior questions included questions on performing oral sex (called oral sex hereafter), open-mouth kissing (French kissing) and vaginal sex. Given the study design to enroll unvaccinated high-risk young adults, we felt it was important to collect data on reasons for declining HPV vaccination. These reasons were also collected in the survey with pre-specified categories for reason as well as an “other reason” free-text response option.

Participants were given a cash incentive ($10) and an incentive gift of $2–$5 value (water-bottle, gum, candy, perfume/cologne, *etc.*). The study was approved by the Institutional Review Board at the Johns Hopkins Bloomberg School of Public Health and Maryland Department of Health and Mental Hygiene.

### 4.2. Laboratory Methods

Oral rinse samples were stored at 4 °C for up to one week until processed. DNA was purified from the oral rinse using a magnetic bead-based automated platform (QIAsymphony SP, Qiagen, Hilden, Germany), as previously described [27]. Purified DNA was then evaluated for 36 different HPV DNA genotypes utilizing PGMY09/11 PCR primer pools and primers for β-globin, followed by reverse line blot hybridization to the Roche^TM^ linear array. Oral HPV types were classified as oncogenic (HPV 16, 18, 31, 33, 35, 39, 45, 51, 52, 56, 58, 59, 68 and 73) and non-oncogenic (HPV 6, 11, 26, 40, 42, 53, 54, 55, 61, 62, 64, 66, 67, 69, 70, 71, 72, 81, 82, 83, 84, 89) [28]. The linear array includes a mixed probe recognizing HPV33, 35, 52 and 58—individuals positive for the probe who were not positive for HPV33, 35 or 59 were considered to have HPV52 infection. As some of these individuals could have been multiply infected the prevalence of HPV52 could be underestimated. Each linear-array included HPV positive DNA (positive control) and negative samples (negative control). Samples were only considered evaluable when it was β-globin positive which demonstrates it has sufficient DNA for the analysis.

### 4.3. Statistical Analysis

At the time of this analysis, study enrollment was complete. Oral HPV prevalence was described overall and when classified as oncogenic, non-oncogenic, or vaccine (HPV 6, 11, 16, 18) types. Multivariate risk factors of oral HPV prevalence were modeled using a log binomial model clustered by person to account for multiple infections. All variables which were significant in univariate analysis, or known to be of importance in the literature, were included in a multivariate model and removed in a stepwise fashion. Because of the co-linearity of sexual behaviors, sexual variables were considered in separate models. When the number of oral sex, vaginal sex, and deep-kissing partners were included in the same model, the association of each variable attenuated due to the collinear nature of these sexual risk factors, but oral sex remained more strongly associated and was therefore retained as the only sexual variable in the final multivariable model. Statistical analyses were conducted using STATA version 12.0 [29].

## 5. Conclusions

This study demonstrated a higher prevalence of oral HPV infection among men than women, even after controlling for sexual behavior. These results are consistent with the hypothesis that differences in the number of oral sexual partners do not completely explain the higher risk of oral HPV infection and HPV-related oropharyngeal cancer in men compared to women.
